# Supplementary material for: 3D genome alterations associated with dysregulated HOXA13 expression in high-risk T-lineage acute lymphoblastic leukemia
Source: Nat Commun. 2021 Jun 17;12:3708. doi: 10.1038/s41467-021-24044-5 (PMC8211852; doi:10.1038/s41467-021-24044-5)
Supplement: Supplementary file 1 — Supplementary Information [file 41467_2021_24044_MOESM1_ESM.pdf]

Supplementary Information

3D Genome Alterations Associated with Dysregulated *HOXA13* Expression in High-Risk T-Lineage Acute Lymphoblastic Leukemia

Yang *et al*.

Supplementary Figures 1-6

Supplementary Table 1

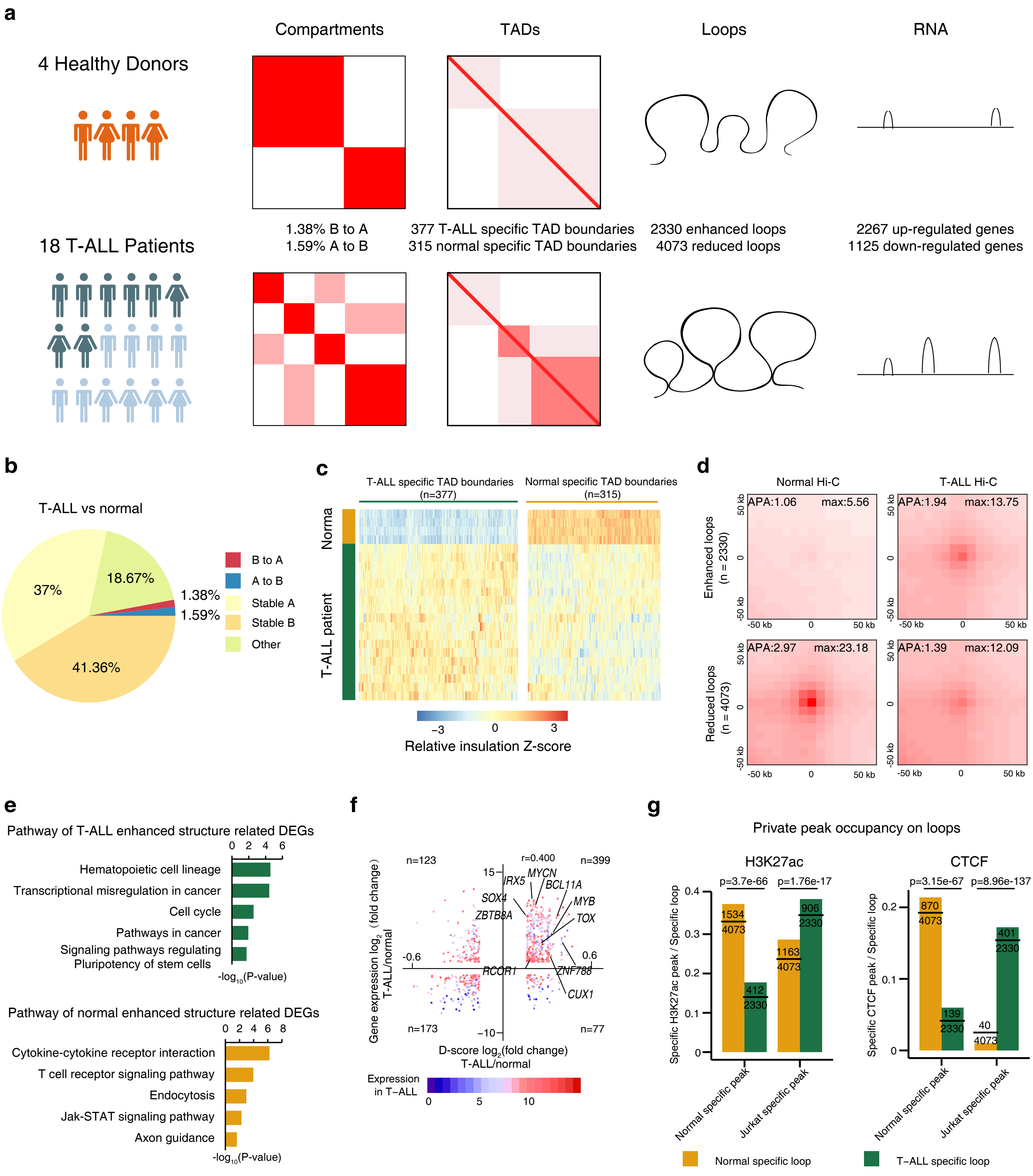

Supplementary Figure 1

Supplementary Fig. 1 Global 3D genome architecture in T-ALLs. a Schematic illustrations of the study design and key findings comparing normal T cell and T-ALL. 22 samples, including 4 healthy donors (orange) and 18 T-ALL patients (8 ETP ALL, dark blue; 10 non-ETP ALL, light blue), are used for integrated genomic and transcriptome analyses. **b** Distributions of genomic regions associated with various compartment changes. **c** Heatmaps showing the relative insulation score of T-ALL specific TAD boundaries (left) and normal specific TAD boundaries (right). **d** APA plots for loops that are enhanced (upper) or reduced (lower) in T-ALLs compared with normal controls. **e** KEGG analysis for enriched pathways in T-ALLs and normal T cells based on DEGs that are associated with chromatin structural changes. **f** Domain score changes are plotted against gene expression changes in all DEGs between T-ALLs and normal T cell controls. **g** The preferential occupancies of specific H3K27ac (left) modification or CTCF binding (right) on loop anchors in normal T cells or T-ALLs. P-values are calculated by one-sided Fisher's exact test. The ratios of cell type-specific modification or binding sites over the total peaks detected are indicated.

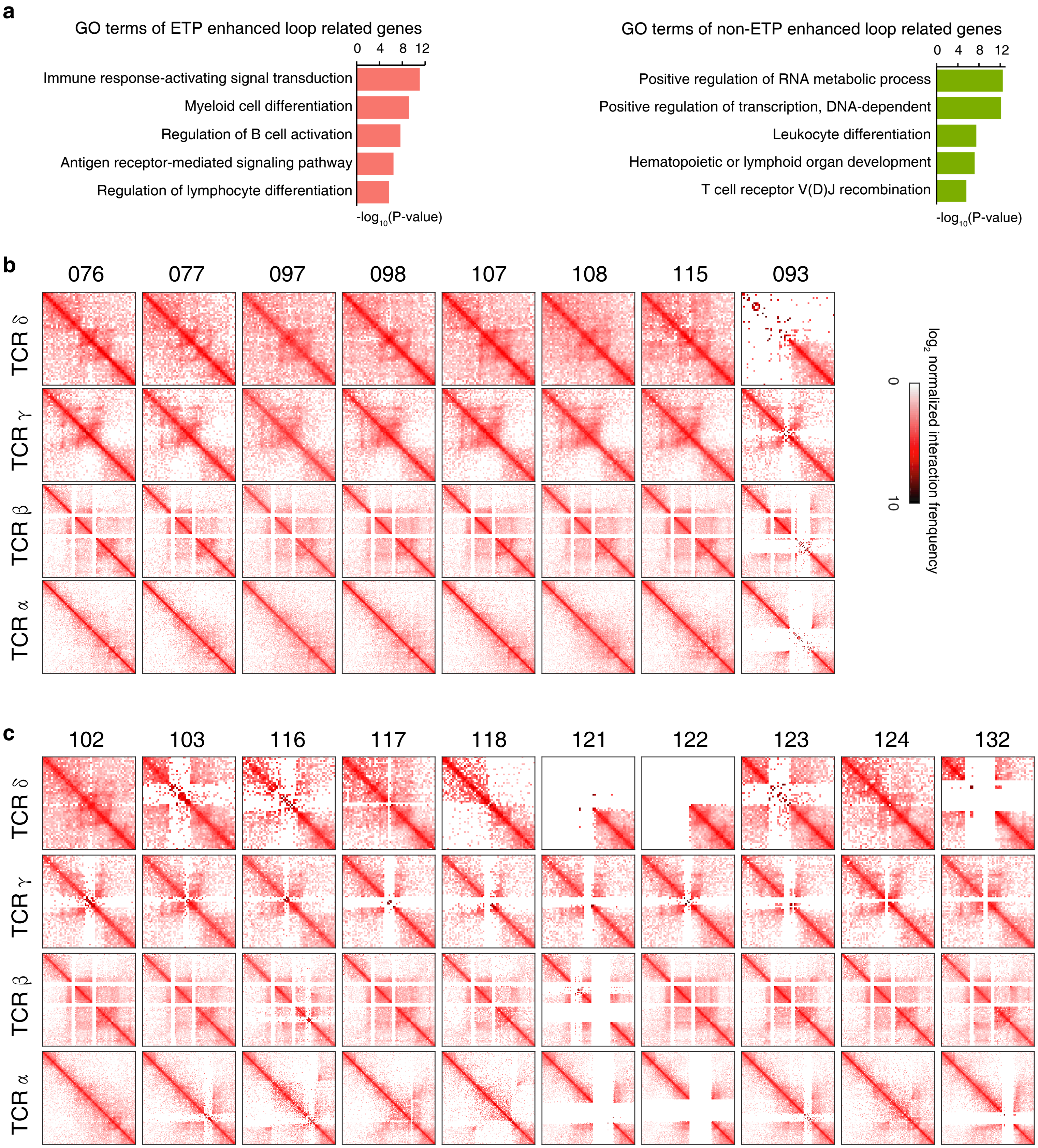

Supplementary Fig. 2 ETP and non-ETP ALLs have different loop structures. **a** GO terms for genes adjacent to enhanced loop anchors in ETP (left) and non-ETP (right). **b** Hi-C contact maps for various TCR regions in 8 ETP cases. **c** Hi-C contact maps for various TCR regions in 10 non-ETP cases.

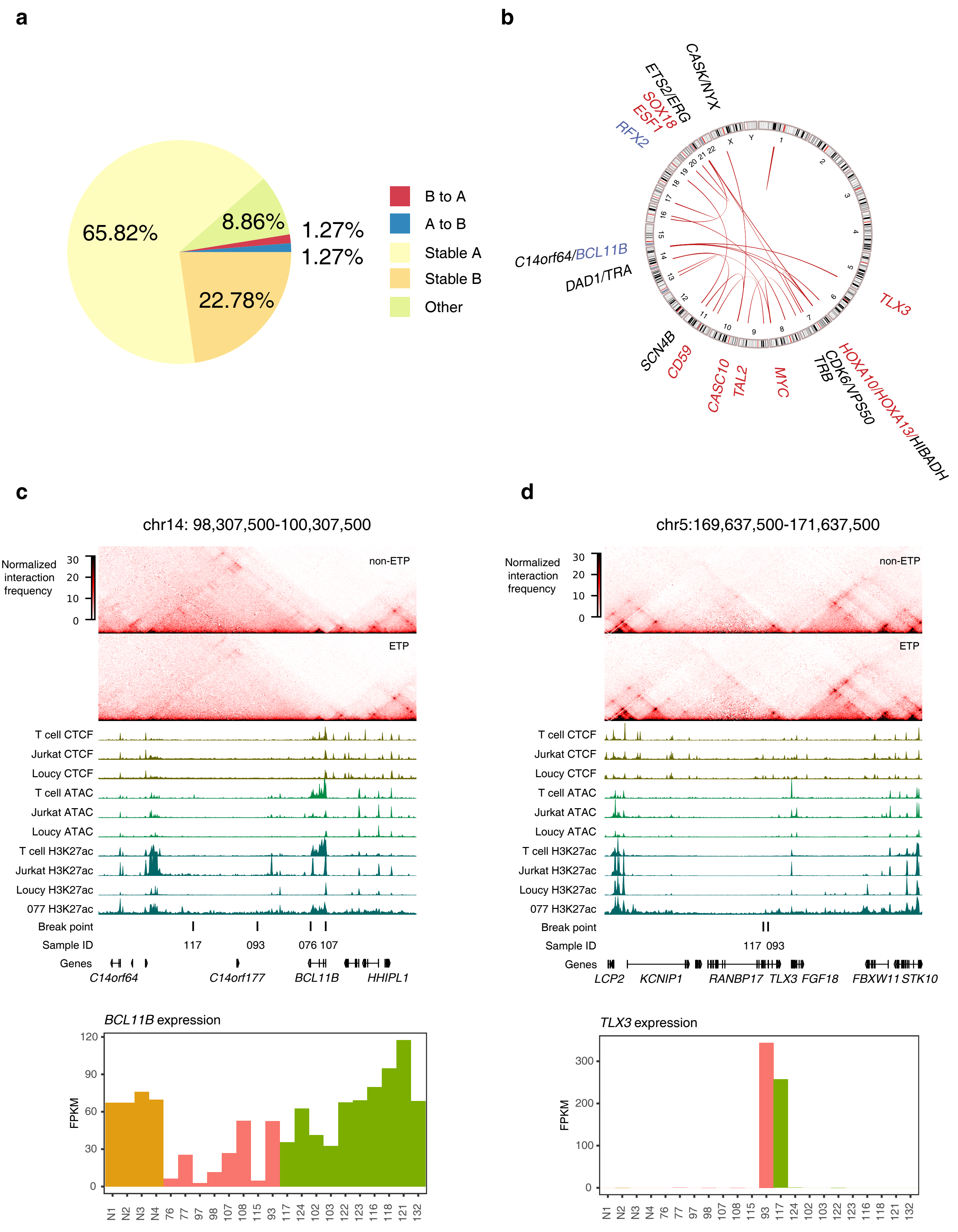

Supplementary Figure 3

Supplementary Fig. 3 Chromosomal rearrangements in T-ALLs. a Distributions of breakpoint locations in various compartments. **b** Previously unrecognized translocation-mediated loops discovered by Hi-C. Genes adjacent to loop anchors with increased and decreased expressions are marked by red and blue, respectively. **c** and **d** Upper: Hi-C contact maps for TADs enclosing the genomic loci of *BCL11B* (**c**) and *TLX3* (**d**) in non-ETP and ETP samples. ChIP-seq tracks for CTCF and H3K27ac and ATAC-seq tracks corresponding to the *BCL11B* (**c**) and *TLX3* (**d**) loci in T cell, Jurkat and Loucy T-ALL cells and case 077 are shown below. Chromosomal breakpoints mapped by this study are marked by vertical lines with case number indicated. Lower: Expression of *BCL11B* and *TLX3* in each sample. *BCL11B-TLX3* translocations lead to ectopic *TLX3* expressions in cases 93 and 117.

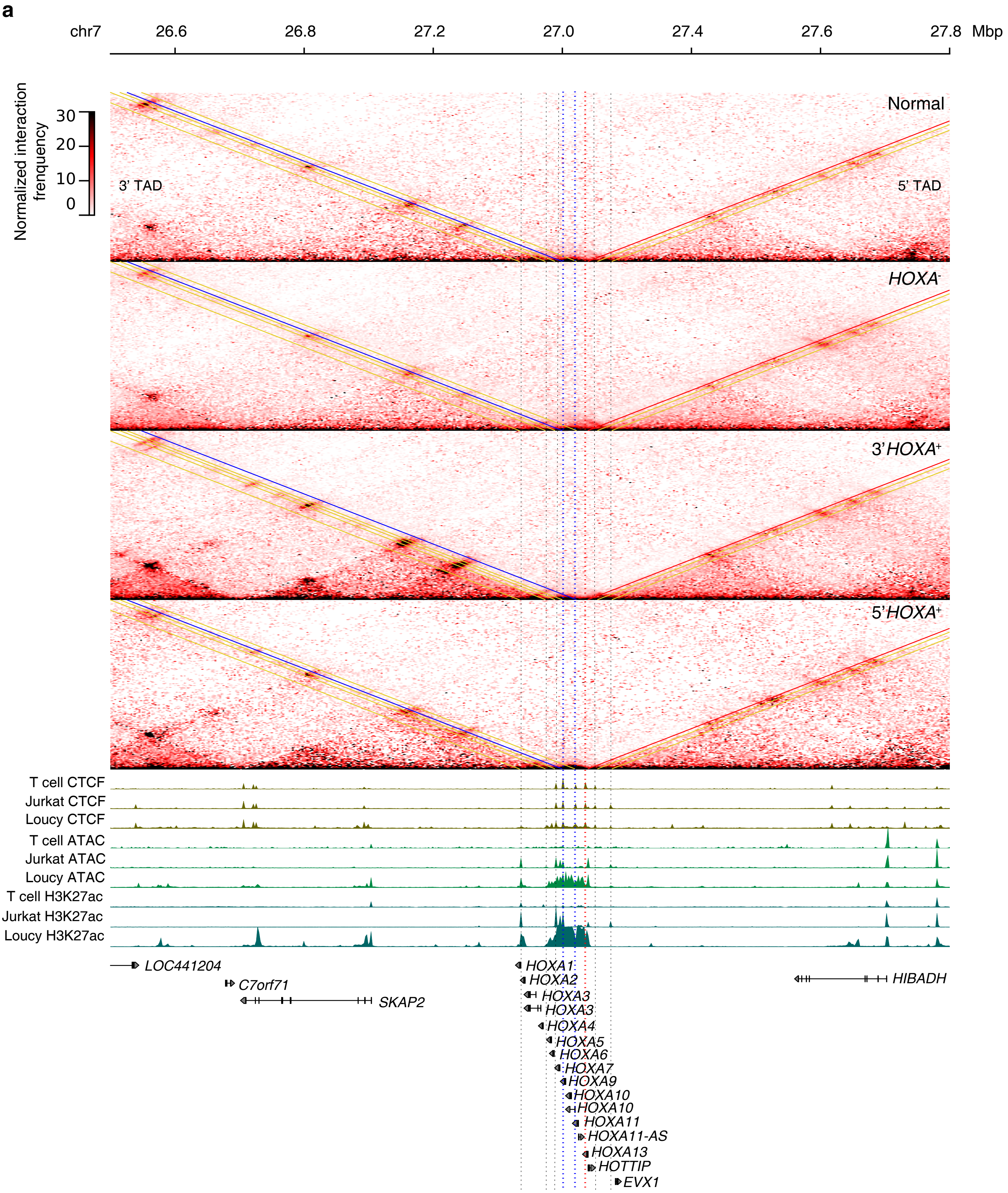

Supplementary Figure 4

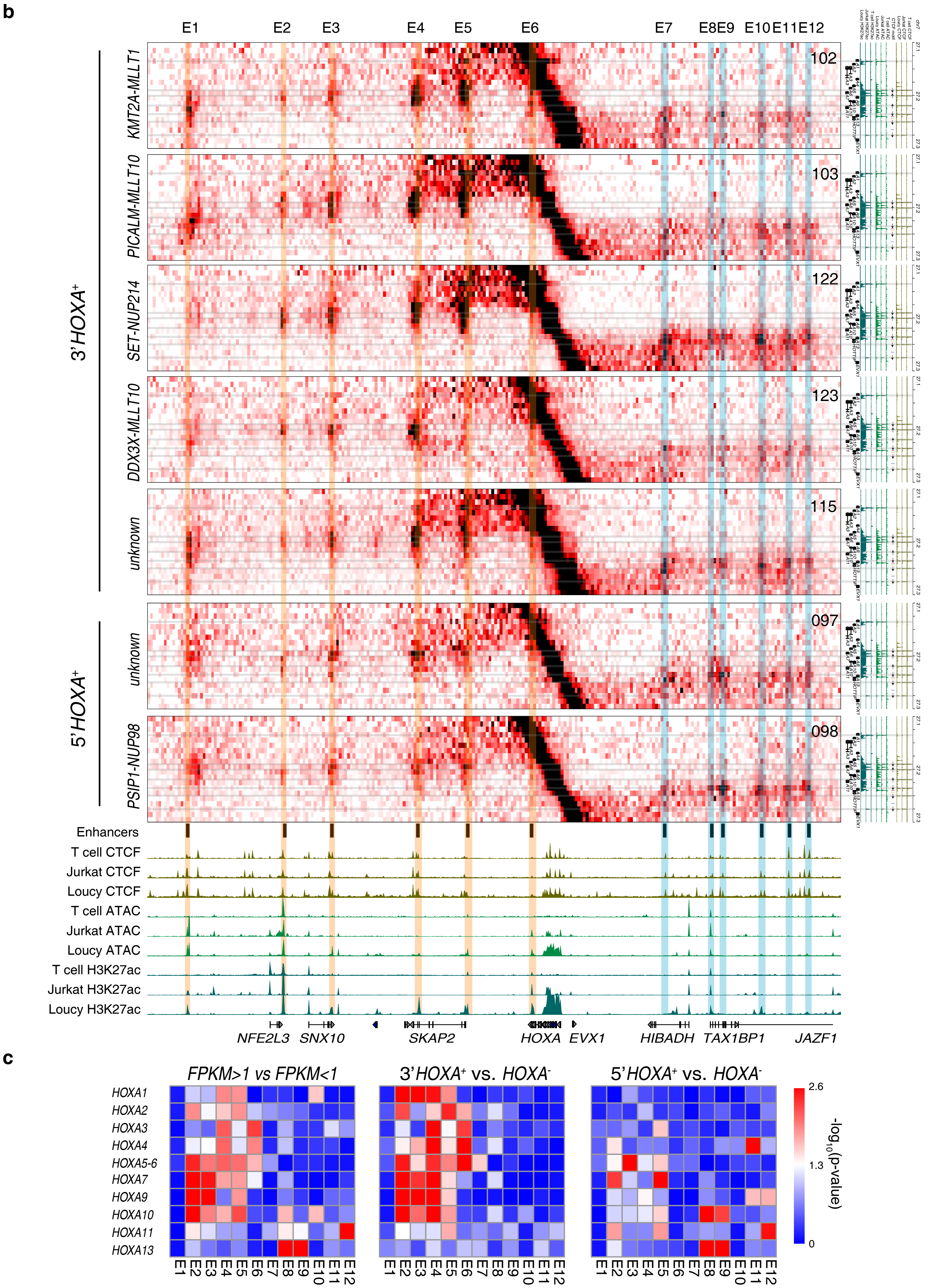

Supplementary Figure 4

d

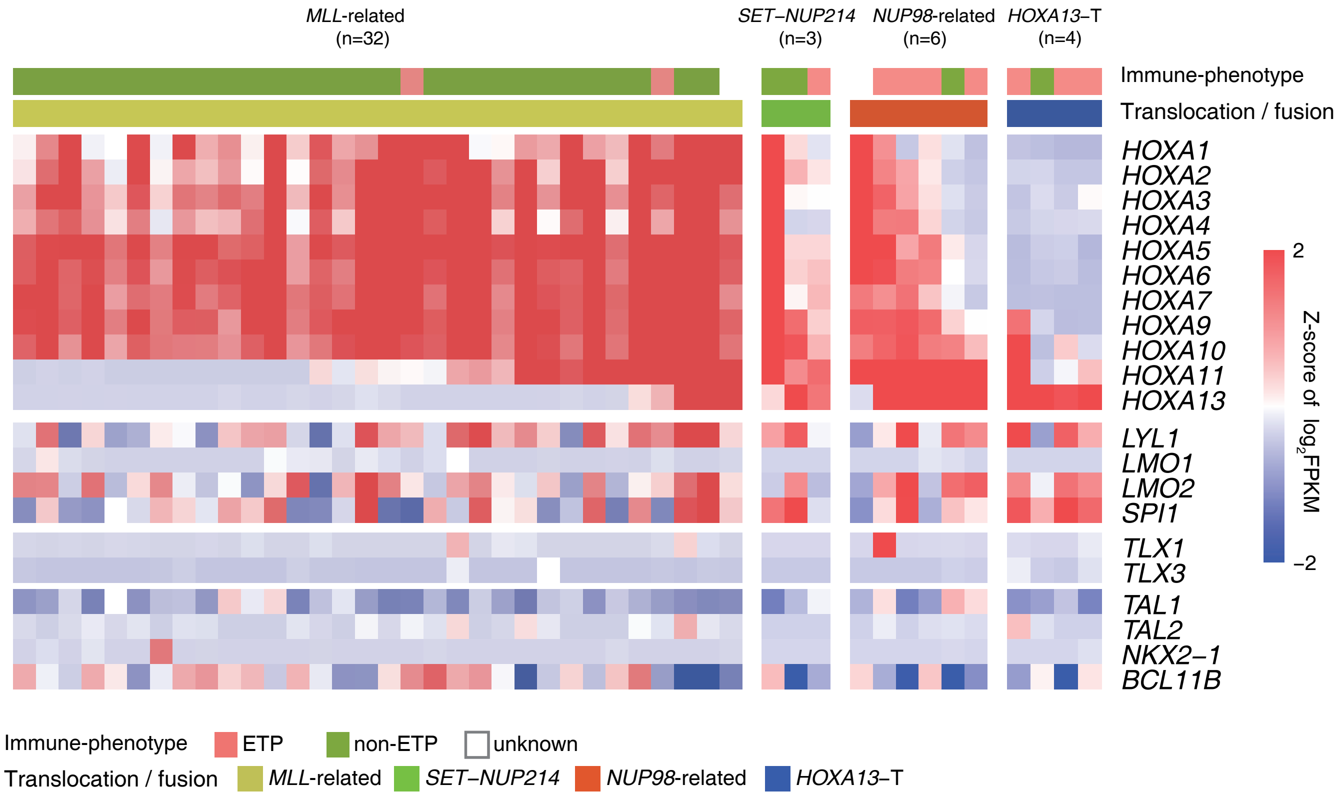

Supplementary Fig. 4 Chromatin interaction profile and expression patterns of the *HOXA* cluster in T-ALLs. a Hi-C heatmaps show the average interaction intensity of normal T cells, *HOXA*$^-$, 3'*HOXA*$^+$ and 5'*HOXA*$^+$ cases in chr7: 25,750,000-28,250,000 (hg19), which includes *HOXA* gene cluster and its 3' and 5' TADs. Vertical black dotted lines mark the CTCF binding sites near the 3' TAD and 5' TAD boundaries; red line marks the 3' boundary of 5' TAD in all samples while blue lines mark the two 5' boundaries of 3' TAD among different samples. ChIP-seq tracks for CTCF and H3K27ac and ATAC-seq tracks corresponding to the *HOXA* cluster in T cell, Jurkat and Loucy T-ALL cells are shown below. **b** Individual Hi-C contact maps between genomic region chr7: 25,750,000-28,250,000 and chr7:27,100,000-27,300,000 for 3'*HOXA*$^+$ and 5'*HOXA*$^+$ cases. The main enhancers are highlighted with orange in the 3' TAD and blue in the 5' TAD. The CTCF sites are heighted with grey horizontal bars. **c** Heatmap showing statistical test conducted by comparing interactome of each *HOXA* gene between its expression as FPKM > 1 and FPKM < 1 (left), 3'*HOXA*$^+$ and *HOXA*$^-$ cases (middle), 5'*HOXA*$^+$ and *HOXA*$^-$ cases (right). P-values are calculated with one-sided t-test. **d** A heatmap shows the profile of leukemogenic transcription factors expression, immune-phenotype and translocation/ fusion of samples in Fig. 4e.

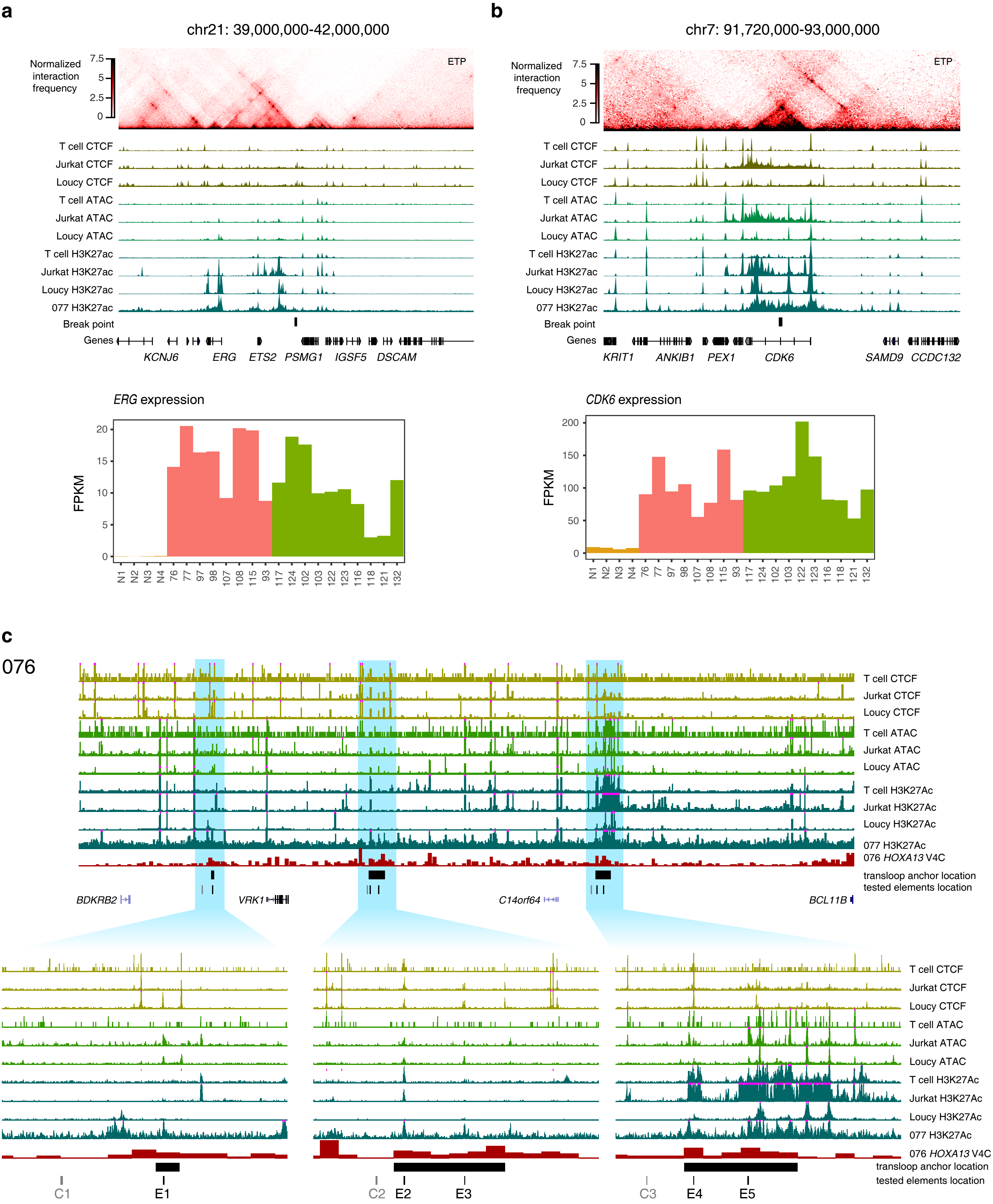

Supplementary Figure 5

d

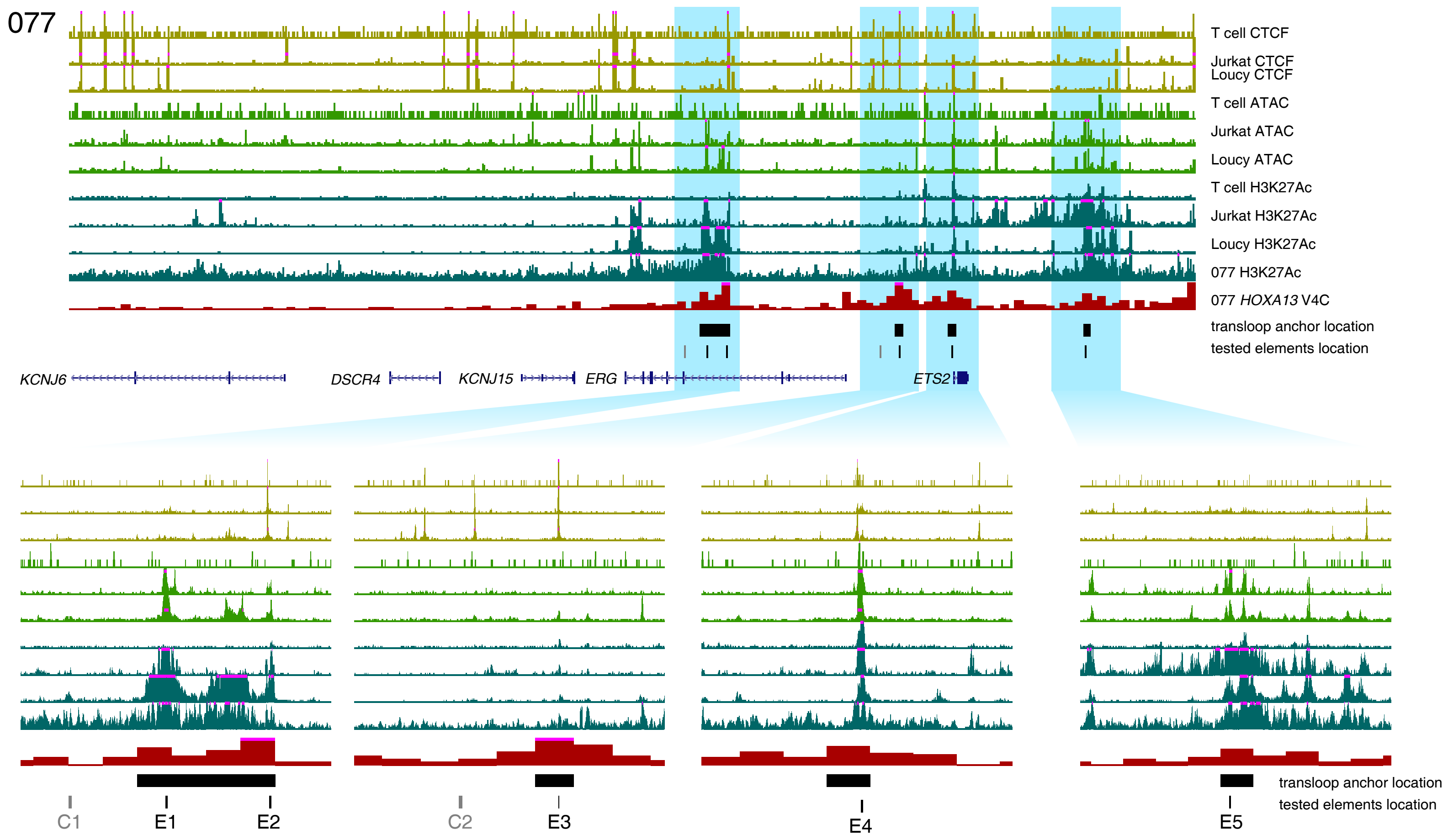

e

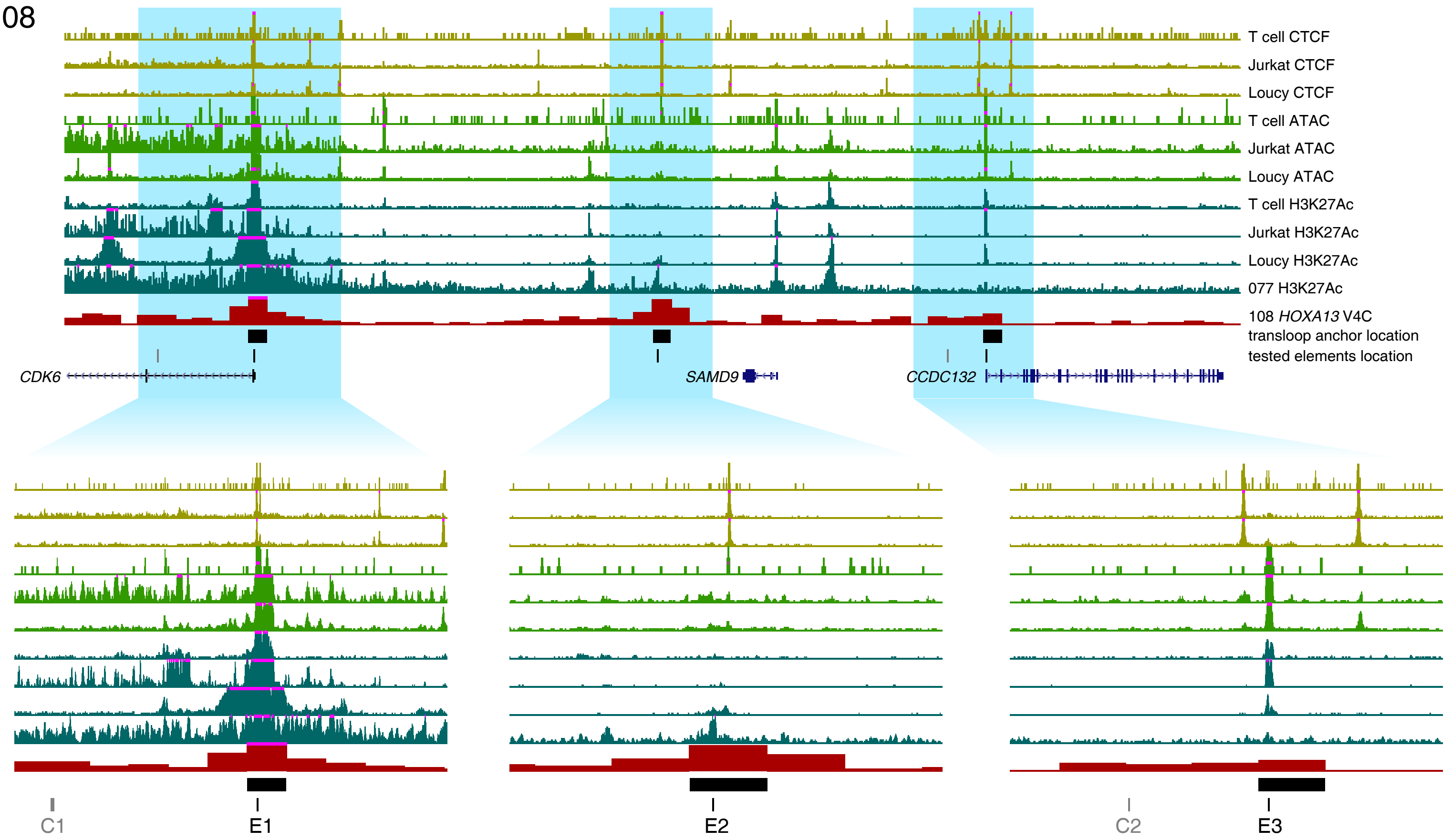

Supplementary Figure 5

f

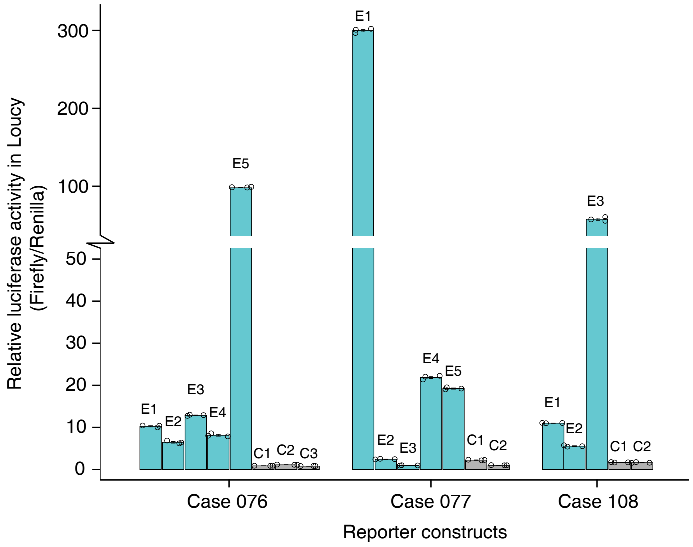

Supplementary Fig. 5 Translocation-mediated enhancer hijacks and ectopic *HOXA* gene expressions in T-ALLs. a and **b** Upper: Hi-C contact maps for TADs enclosing the genomic loci of *ERG* (a) and *CDK6* (b) genes. Lower: The expression levels of *ERG* and *CDK6* genes in each sample. **c-e** CTCF binding, ATAC data and enhancer-histone marks (H3K27ac) overlapping the translocated regions of case 076, 077 and 108, respectively, in T cell, Jurkat and Loucy T-ALL cell lines as well as case 077. Red tracks are visual 4C plots of *HOXA13*. The locations of loop anchors and predicted enhancers are also indicated in panels. **f** Luciferase reporter activities for regions encompassed within the predicted enhancers and control regions indicated in panel Fig.5**a-c** compared to empty vector in Loucy cell line (n=3 per group). Data are represented as mean ± SD.

a

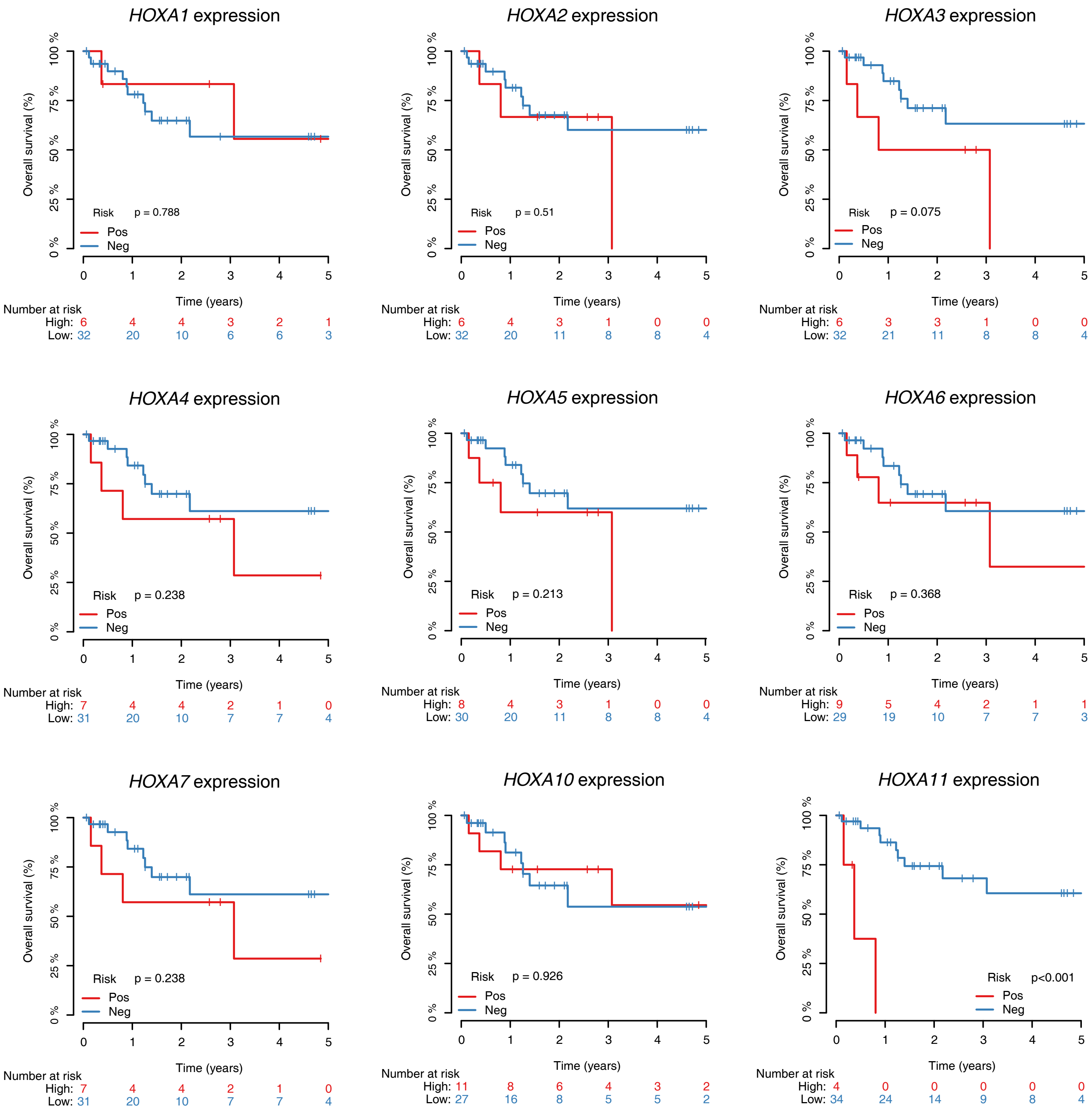

Supplementary Figure 6

b

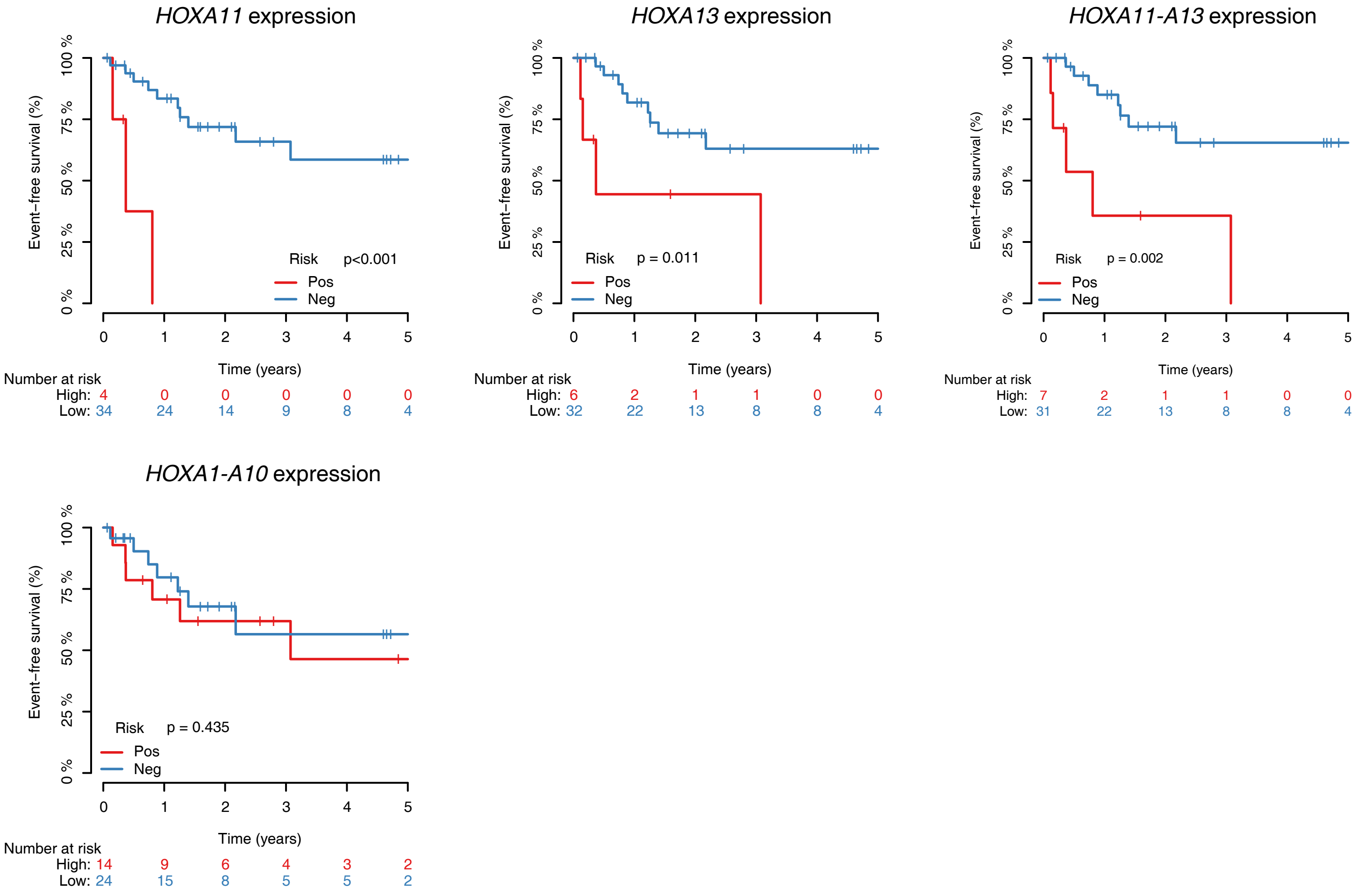

Supplementary Fig. 6 Ectopic *HOXA11-A13* expressions are correlated with poor outcomes in pediatric and young adult T-ALLs. a Kaplan-Meier overall survival curves of pediatric and young adult patients with (red) or without (blue) *HOXA1* to *HOXA11*. **b** Kaplan-Meier event free survival curves of pediatric and young adult patients with (red) or without (blue) *HOXA11* or *HOXA13* expression, *HOXA11-A13* expression, *HOXA* expression excluding *HOXA11* or *A13* expression (*HOXA1-A10*). *P* values are calculated by the two-sided log-rank test.

Supplementary Table 1 | List of primers for Luciferase constructs

Case076-E1	chr14:97,042,853-97,043,853	Forward	GTAGTTACAGATCAGTGAGACCTGGT
		Reward	GCTCTGAGCCTCAAGATCTTTCTCA
Case076-E2	chr14:97,684,968-97,685,899	Forward	CTGAGCTCGCTAGCCTCGACCCAGTGACGACTGGATTCGG
		Reward	ACCCTCTAGTGTCTAAGCTCGCCCAGACCGCCGCGTCCCCA
Case076-E3	chr14:97,717,757-97,718,757	Forward	ACTTTTTAGTAACCAGGTCCAGAACG
		Reward	GCACCGCCTCTCTGCTTTCTGCCTGA
Case076-E4	chr14:98,604,563-98,605,563	Forward	GCCTTATGTGTTTGAAAATGATCTCT
		Reward	GTTCTGTACTAATGGATCTTTCCAACT
Case076-E5	chr14:98,633,808-98,634,809	Forward	CTGAGCTCGCTAGCCTCGAAGACCTAGGATGGGCTGGCCAC
		Reward	ACCCTCTAGTGTCTAAGCTCCTCTCCCATCTTTTAAAGTTCT
Case076-C1	chr14:97,000,000-97,001,000	Forward	CCTGCCTTAGTCTCCTGAGTAGCTGG
		Reward	TCATAAGTAAAAGGCTGGAAGTCTC
Case076-C2	chr14:97,670,000-97,671,000	Forward	AGCCAGTGTTCCCATGTTTAC
		Reward	CTTCATGCTAGATACAACAGGGATC
Case076-C3	chr14:98,580,000-98,581,000	Forward	ACATCACTGATTTTAAGCC
		Reward	CACAGGCCTGGGAAGAGTTCCTGTT
Case077-E1	chr21:39,847,570-39,848,570	Forward	GCAAACAAAGCCCTAAGTGTCGCTG
		Reward	CGTTGTTGTTGGTGCACATCTGC
Case077-E2	chr21:39,878,343-39,879,285	Forward	CTGAGCTCGCTAGCCTCGAAAAGCCTCACGGTCCCTTCCTT
		Reward	ACCCTCTAGTGTCTAAGCTGAATATTGGCACAAATGAATTAA
Case077-E3	chr21:40,105,505-40,106,505	Forward	TGAGGATTGCACAAAAACTAGAAATTCC
		Reward	CCCGACTCTCCTCGCACCTTCACA
Case077-E4	chr21:40,177,355-40,178,223	Forward	CTGAGCTCGCTAGCCTCGAATTCCAAAGGCAGGTTTGGCG
		Reward	ACCCTCTAGTGTCTAAGCTCGCGGAACTCAGACGCGCGCCCC
Case077-E5	chr21:40,105,505-40,106,505	Forward	TCTTCTTCCTAAAGACTTTCCAGGAC
		Reward	AGGGGCTCTGGAGCCACAAGGCA
Case077-C1	chr21:39,820,000-39,821,000	Forward	CCATTCTCACATCTGGCAAAGCA
		Reward	ATCTATACATGCTAACTAAGGCAGC
Case077-C2	chr21:40,080,000-40,081,000	Forward	GTGAATATGAACAGAAATGTGTA
		Reward	CCAGTCATTACTCATGCCCTGGGAC
Case108-E1	chr7:92,462,003-92,462,962	Forward	CTGAGCTCGCTAGCCTCGATCTTTCAAAAATTCCCCAACGGC
		Reward	ACCCTCTAGTGTCTAAGCTGTAATCGTGTCTGTGTTGAGGA
Case108-E2	chr7:92,682,625-92,683,374	Forward	CTGAGCTCGCTAGCCTCGAAATAACTTCCTCCATTCTCTCT
		Reward	ACCCTCTAGTGTCTAAGCTAGTTGTGGAATCGACTACTCTG
Case108-E3	chr7:92,861,082-92,862,082	Forward	GAGTTTGATCTTTCGCTCAACGCCT
		Reward	CAGGGTATCCTGTCTTTCCCTGGGCC
Case108-C1	chr7:92,410,000-92,411,000	Forward	AGAGGCAGAGCTTGCAGTGAG
		Reward	CATAGAACTTCCTGTGAATGGGT
Case108-C2	chr7:92,840,000-92,841,000	Forward	ATGGTTTGCTCAGTAATGGCCTA
		Reward	GCTGACAACATGAAGGGTCCCTTC
