# Supplementary material for: 3D genome alterations associated with dysregulated HOXA13 expression in high-risk T-lineage acute lymphoblastic leukemia
Source: Nat Commun. 2021 Jun 17;12:3708. doi: 10.1038/s41467-021-24044-5 (PMC8211852; doi:10.1038/s41467-021-24044-5)
Supplement: Supplementary file 3 — Description of Additional Supplementary Files [file 41467_2021_24044_MOESM3_ESM.pdf]

Description of Additional Supplementary Files

File name: Supplementary Data 1
Description: Quality control of Hi-C data.

File name: Supplementary Data 2
Description: Differential analysis of Hi-C and RNA-seq data between T-ALL and normal T cell. DESeq2 estimate variance-mean dependence based on RNA-Seq count data and test for DEGs based on negative binomial distribution.

File name: Supplementary Data 3
Description: Differential analysis of Hi-C and RNA-seq data between ETP-ALL and non-ETP-ALL. DESeq2 estimate variance-mean dependence based on RNA-Seq count data and test for DEGs based on negative binomial distribution.

File name: Supplementary Data 4
Description: Overview of predicted translocation events.

File name: Supplementary Data 5
Description: Overview of detected trans-loop.

File name: Supplementary Data 6
Description: Patient and T cell donor information
