# Supplementary material for: 3D genome alterations associated with dysregulated HOXA13 expression in high-risk T-lineage acute lymphoblastic leukemia
Source: Nat Commun. 2021 Jun 17;12:3708. doi: 10.1038/s41467-021-24044-5 (PMC8211852; doi:10.1038/s41467-021-24044-5)
Supplement: Supplementary file 10 — Reporting Summary [file 41467_2021_24044_MOESM10_ESM.pdf]

nature research

Corresponding author(s): Hong Wu
Last updated by author(s): Mar 20, 2021

Reporting Summary

Nature Research wishes to improve the reproducibility of the work that we publish. This form provides structure for consistency and transparency in reporting. For further information on Nature Research policies, see our Editorial Policies and the Editorial Policy Checklist.

Statistics

For all statistical analyses, confirm that the following items are present in the figure legend, table legend, main text, or Methods section.

n/a	Confirmed	
☐	☒	The exact sample size (n) for each experimental group/condition, given as a discrete number and unit of measurement
☐	☒	A statement on whether measurements were taken from distinct samples or whether the same sample was measured repeatedly
☐	☒	The statistical test(s) used AND whether they are one- or two-sided<br>*Only common tests should be described solely by name; describe more complex techniques in the Methods section.*
☐	☒	A description of all covariates tested
☒	☐	A description of any assumptions or corrections, such as tests of normality and adjustment for multiple comparisons
☐	☒	A full description of the statistical parameters including central tendency (e.g. means) or other basic estimates (e.g. regression coefficient) AND variation (e.g. standard deviation) or associated estimates of uncertainty (e.g. confidence intervals)
☐	☒	For null hypothesis testing, the test statistic (e.g. F, t, r) with confidence intervals, effect sizes, degrees of freedom and P value noted<br>*Give P values as exact values whenever suitable.*
☒	☐	For Bayesian analysis, information on the choice of priors and Markov chain Monte Carlo settings
☐	☒	For hierarchical and complex designs, identification of the appropriate level for tests and full reporting of outcomes
☐	☒	Estimates of effect sizes (e.g. Cohen's d, Pearson's r), indicating how they were calculated

Our web collection on statistics for biologists contains articles on many of the points above.

Software and code

Policy information about availability of computer code

Data collection	No special software was used.
Data analysis	TopHat (v2.1.0), HTSeq (v0.6.0), DESeq2 (v1.24.0), BEDTools (v2.27.1), UCSC Genome Browser utility, Bowtie2 (v2.3.5), SAMtools (v1.9), MACS2 (v2.2.5), FIMO (v5.0.2), ChIA-PET2 (v0.9.2), HiC-Pro (v2.11.1), Juicer, Insulation score, HiCDB, GREAT (v3.0.0), DAVID 6.8, pyGenomeTracks (v3.1.2), hic_breakfinder , Juicebox (v1.9.8), Matlab (R2018a)<br>custom codes: HiCpipe (https://github.com/ChenFengling/HiCpipe)

For manuscripts utilizing custom algorithms or software that are central to the research but not yet described in published literature, software must be made available to editors and reviewers. We strongly encourage code deposition in a community repository (e.g. GitHub). See the Nature Research guidelines for submitting code & software for further information.

Data

Policy information about availability of data

All manuscripts must include a data availability statement. This statement should provide the following information, where applicable:

- Accession codes, unique identifiers, or web links for publicly available datasets
- A list of figures that have associated raw data
- A description of any restrictions on data availability

The raw data of Hi-C, RNA-seq, ATAC-seq and H3K27ac-ChIP-seq have been deposited in the Genome sequence Archive in BIG Data Center, Beijing Institute of Genomics (BIG), Chinese Academy of Science, under accession number HRA000113 (https://bigd.big.ac.cn/gsa-human). The processed data have been deposited in GEO, under accession number GSE146901. GEO accession codes of the published data used in this study are as follows: CTCF ChIP-seq of CD4+ T cell and Jurkat cell line, GSE12889; CTCF ChIP-seq of Loucy cell line, GSE123214; ATAC-seq of CD4+ T cell, GSE87254; ATAC-seq of Jurkat cell line, GSE115438; H3K27ac ChIP-seq of CD4 + T cell, GSE122826; H3K27ac ChIP-seq of Jurkat cell line, GSE68978; H3K27ac ChIP-seq of Loucy cell line, GSE74311; RNA-Seq of Loucy cell line, GSE100694; RNA-

seq of T cell development, GSE69239.

Field-specific reporting

Please select the one below that is the best fit for your research. If you are not sure, read the appropriate sections before making your selection.

- [x] Life sciences
- [ ] Behavioural & social sciences
- [ ] Ecological, evolutionary & environmental sciences

For a reference copy of the document with all sections, see nature.com/documents/nr-reporting-summary-flat.pdf

Life sciences study design

All studies must disclose on these points even when the disclosure is negative.

Sample size	Sample size were determined in order to obtain more than 10 T-ALL samples and almost equal sample size in T-ALL subgroups.
Data exclusions	There is no data that were excluded from the analyses.
Replication	No replication was performed in obtaining Hi-C data and RNA-seq data; for each patient biopsy was sequenced once.
Randomization	Randomization was relevant to the study, because the difference between healthy and disease was assessed.
Blinding	The investigators were not blinded to sample group allocation, because the difference between healthy and disease was assessed. Sample group assignments were further ensured using Principal Component Analysis on all relevant sequencing data.

Reporting for specific materials, systems and methods

We require information from authors about some types of materials, experimental systems and methods used in many studies. Here, indicate whether each material, system or method listed is relevant to your study. If you are not sure if a list item applies to your research, read the appropriate section before selecting a response.

Materials & experimental systems

n/a	Involved in the study	
[ ]	[x]	Antibodies
[ ]	[x]	Eukaryotic cell lines
[x]	[ ]	Palaeontology and archaeology
[x]	[ ]	Animals and other organisms
[ ]	[x]	Human research participants
[x]	[ ]	Clinical data
[x]	[ ]	Dual use research of concern

Methods

n/a	Involved in the study	
[ ]	[x]	ChIP-seq
[x]	[ ]	Flow cytometry
[x]	[ ]	MRI-based neuroimaging

Antibodies

Antibodies used	Anti-H3K27ac (Abcam ab4729, lot #: GR3251519-2, 2ug antibody for 25ug chromatin);<br>IgG (Abcam ab171870, lot#: GR288638-2, use a concentration of 1ug/ml)
Validation	Validation of antibodies is ensured by commercial manufacture for the application used.<br>For H3K27ac antibody from Abcam, the datasheet for validation is available at https://www.abcam.com/histone-h3-acetyl-k27-antibody-chip-grade-ab4729.pdf<br>For isotype control IgG from Abcam, the datasheet for validation is available at https://www.abcam.com/rabbit-igg-polyclonal-isotype-control-chip-grade-ab171870.pdf

Eukaryotic cell lines

Policy information about cell lines

Cell line source(s)	Loucy cells are from ATCC (https://www.atcc.org/products/all/CRL-2629.aspx);<br>Jurkat cells are from China Center for Type Culture collection (CCTCC, http://www.cellresource.cn/contact.aspx) and the resource code is 3131C0001000700123;<br>KE-37 cells are from DSMZ, DSMZ no.: ACC 46, RRID: CVCL_1327.
Authentication	The identity of the cell line was authenticated with STR profiling by the provider, all the results can be viewed on the website.
Mycoplasma contamination	Cell line used in this study tested negative for mycoplasma contamination.

Commonly misidentified lines (See ICLAC register)	No commonly misidentified lines were used in the study.

Human research participants

Policy information about studies involving human research participants

Population characteristics	Relevant co-variate data on patients and healthy donors can be found in Supplementary Data 6.
Recruitment	All primary bone marrow aspirates were taken from routine diagnostic specimens after informed consent of the patients. Healthy T cells were taken from peripheral blood of healthy donors after obtained the consent. Leukemia samples have been selected for two specific sub-types but potential biased are discussed in Figure 1 and 2 combining expression and chromatin interaction information at TCR locus.
Ethics oversight	Samples were collected by Peking University People's Hospital with informed consent and approved and analyzed under the supervision of the Ethics Committee of Peking University People's Hospital. Written informed consent was obtained in accordance with Chinese legislation.

Note that full information on the approval of the study protocol must also be provided in the manuscript.

ChIP-seq

Data deposition

☒ Confirm that both raw and final processed data have been deposited in a public database such as GEO.

☒ Confirm that you have deposited or provided access to graph files (e.g. BED files) for the called peaks.

Data access links<br>*May remain private before publication.*	https://www.ncbi.nlm.nih.gov/geo/query/acc.cgi?acc=GSE146901<br>https://bigd.big.ac.cn/gsa-human/browse/HRA000113
Files in database submission	Fastq and bigwig files of ChIP-seq data<br>ChIP_H3K27ac_077_rep1_R1.fq.gz<br>ChIP_H3K27ac_077_rep1_R2.fq.gz<br>ChIP_H3K27ac_077_rep2_R1.fq.gz<br>ChIP_H3K27ac_077_rep2_R2.fq.gz<br>ChIP_IgG_077_R1.fq.gz<br>ChIP_IgG_077_R2.fq.gz<br>077_H3K27ac.bw<br>ChIP_H3K27ac_KE37_R1.fq.gz<br>ChIP_H3K27ac_KE37_R2.fq.gz<br>ChIP_IgG_KE37_R1.fq.gz<br>ChIP_IgG_KE37_R2.fq.gz<br>KE37_H3K27ac.bw
Genome browser session (e.g. UCSC)	https://genome.ucsc.edu/s/ChenFengling/TALL

Methodology

Replicates	2 replicates for H3K27ac ChIP-Seq of patient 077, no replicate for H3K27ac ChIP-Seq of KE37.
Sequencing depth	35M reads for H3K27ac ChIP-Seq of patient 077; 11M reads for H3K27ac ChIP-Seq of KE37 cell line;
Antibodies	H3K27ac antibody (Abcam ab4729, lot #: GR3251519-2) and IgG was used as a negative control (Abcam ab171870, lot#: GR288638-2)
Peak calling parameters	Default parameters of MACS2
Data quality	Reads with a Phred quality score of <20 were removed. Non-unique reads were removed by SAMtools. Quality were accessed by deeptools and UCSC Genome Browser.
Software	Bowtie2, SAMtools, MACS2, UCSC Genome Browser utility, ChIPseeker
